# Free radical scavenging capacity and antioxidant activity of methanolic and ethanolic extracts of plum (Prunus domestica L.) in both fresh and dried samples

**Published:** 2014

**Authors:** Amin Morabbi Najafabad, Rashid Jamei

**Affiliations:** 1 *Department of Biology, Faculty of Science, Urmia University, Urmia, I. R. Iran*

**Keywords:** *Antioxidantactivity*, *Correlation*, *Phenoliccompounds*, *PrunusdomesticaL*.

## Abstract

**Objectives**: Consumption of fruits, such as plums and prunes, is useful in treating blood circulation disorder, measles, digestive disorder, and prevention of cancer, diabetes, and obesity. The paper presents a description of antioxidant and antiradical capacity of plum (*Prunus domestica* L.) in both fresh and dried samples.

**Materials and Methods: **Samples were mixed with methanol and ethanol (as solvents) and were extracted on magnetic shaker, separately. The experiments were carried out to measure the Total Phenolic Content (TPC), Total Flavonoid Content (TFC), Total Antioxidant Capacity (TAC), Reducing Power Assay (RPA), Chain Breaking Activity (CBA), and quantity of Malondialdehyde (MDA), 2,2-Diphenyl-1-Picrylhydrazyl (DPPH),Nitric Oxide (NO),Hydrogen peroxide (H_2_O_2_) and superoxide(O_2_^-^) radicals inhibition.

**Results: **The results showed that the highest values for the TPC, TFC,TAC, RPA, CBA, DPPH, and NO were related to ethanolic extractsof dried sample which showed statistically significant differences (p<0.01 and p<0.0001), while the maximum values for the H_2_O_2_ and O_2_^-^were related to ethanolic extracts of fresh sample. The correlations data were analyzed among all parameters and the TPC and TFC had a significant correlation (r^2^=0.977). Moreover, it was found that methanol was more successful in extraction procedure than ethanol (p<0.01).

**Conclusion: **Findings suggest that the fresh samples are more successful in collecting oxygen free radicals such as superoxide (O_2_^-^) and peroxy radicals (ROO^.^) than dried.

## Introduction

More than 100 species of plum are cultivated in the temperate zones throughout the world since prehistoric times. Commonly, dried plums are called prunes (Jabeen and Aslam, 2001 ▶). Prunes are considered as healthy food because of lower fat contents and contain considerable amount of important nutrients such as carbohydrates, vitamins, and minerals. Prunes and prune products also possess medicinal value. Consumption of fruits, such as plums and prunes, is useful in treating blood circulation disorder, measles, digestive disorder (Li, 2008 ▶), and in prevention of cancer, diabetes, and obesity. Plum fruits also contain copious amounts of natural phenolic phytochemicals, such as flavonoids, phenolic acids, anthocyanins, and other phenolics, which may function as effective natural antioxidants in our daily diet (Kristl et al., 2011 ▶). 

Recent studies demonstrated that the cancer preventing actions of prunes are associated with its polyphenolic contents and antioxidant activity, which have inhibitory effects on mutagenesis and carcinogenesis (Jabeen and Aslam, 2001 ▶). Caffeoylquinic acids, hydorxycinnamic acids, protocatechuic acid, coumarins, lignins, and flavanoids present in prunes have high antioxidant activity (Kikuzaki et al., 2004 ▶). There has been strong evidence indicating that free radicals cause oxidative damage to lipids, proteins, and nucleic acids (Shui and Leong, 2004 ▶). 

A free radical is defined as any atom or molecule possessing unpaired electrons (Umamaheswari and Chatterjee, 2008 ▶). In living systems, free radicals are generated as part of the body's normal metabolic process (Saha et al., 2008 ▶). Antioxidants fight free radicals and protect us from various diseases. Though antioxidant enzymes such as superoxide dismutase, catalase, and glutathione peroxidase play an important role in scavenging free radicals and oxidants, these defense mechanisms are not adequate. Consequently, cellular macromolecules are easily subject to oxidative damage (Bergendi et al., 1999 ▶). Several studies have revealed that a major part of the antioxidant activity may be from compounds such as flavonoids, flavones, isoflavones, anthocyanin, catechin, and other phenolic compounds (Kähkönen et al., 1999 ▶). 

Phenolic compounds are secondary metabolites, widely distributed in plants. They are important components of many fruits and vegetables not only for their major influence on sensory qualities of the fruit (color, flavor, and taste), but also for their antioxidant, anticarcinogenic, antimicrobial, antiallergic, antimutagenic, and anti-inflammatory properties (Alesiani et al., 2010 ▶). Therefore, the role of fruits and vegetables in disease prevention is partly associated with the antioxidant properties of their constituent phenolics (Scalbert and Williamson, 2000 ▶). Recently, phenolics have been considered as powerful antioxidants *in vitro* and proved to be more potent antioxidants than vitamins C and E and carotenoids (Rice-Evans et al., 1996 ▶). There are numerous references in the literature indicating the antioxidant activity of various cultivars of plum. However, there is little information concerning the evaluation of antioxidant activity and antiradical capacity of plum in both dried and fresh conditions that is the process of extraction by different solvents. Therefore, the objective of this study was to provide free radical scavenging capacity and antioxidant activity of plum in both dried and fresh samples.

## Materials and Methods

**Chemicals and reagents**

All chemicals and reagents were purchased from Sigma Chemical Co. (St. Louis, MO, USA).

**Plant materials and extraction procedure**

Plums were collected from the Sadaghyan village of Salmas located in West Azarbaijan province in northwestern Iran. The scientific name of the plums was diagnosed by the Agricultural Research Center of West Azarbaijan province. Some of the plums were kept in –80 °C until experiments and part of them was dried in the sun (with daily average temperature of 33 °C) for 20 days.

The kernel of plums was removed in both dried and fresh samples and their flesh (50 g) turned into tiny pieces. The homogeneous of plums content were transferred to volumetric flasks and were mixed with 99.5% methanol (250 ml) as solvent. Above steps were repeated again with the replacement of 99.5% ethanol instead of methanol. The volumetric flasks were extracted on magnetic shaker for 3 hours. The solutions were filtered through Whatman No.1 filter paper to obtain a clear supernatant and then centrifuged at 4000 g for half an hour. The solutions were sealed and stored at 4 °C until experiments.

**Determination of total phenolic content (TPC)**

The TPC was determined using the Folin-Ciocalteau reagent according to the method of Horwits (1984) ▶. The absorbance of the solution was determined at 750 nm using a spectrophotometer (Biowave, S2100, UK) and compared with gallic acid equivalents calibration curve. The TPC was expressed as mg gallic acid equivalents (GAE) /100 g extract.

**Determination of total flavonoid content (TFC)**

The TFC was measured by a colorimetric assay developed by Zhishen et al. (1999) ▶. Absorbance of the mixture was determined at 510 nm versus prepared water blank. Quercetin was used as standard for the calibration curve. The TFC was expressed as mg quercetin equivalents (QE) /100 g extract.

**Evaluation of total antioxidant capacity (TAC)**

The TAC was evaluated by the method of Prieto et al. (1999) ▶. The TAC was expressed as mg equivalents of α-tocopherol using the standard tocopherol graph.

**Reducing power assay (RPA)**

This was carried out as described previously by Yildrim et al. (2001) ▶. 2.5 ml of sample were mixed with 2.5 ml of sodium phosphate buffer (0.2 M pH 6.6) and 2.5 ml of 1% potassium ferricyanide. The mixture was incubated at 50 °C for 30 min. Afterwards, 2.5 ml of 10% trichloroacetic acid (w/v) was added and the mixture was centrifuged at 2000 g for 10 min (BHG 1100 centrifuge, Rotina 35R, Hettich, Germany). The upper layer (2.5 ml) was mixed with deionized water (2.5 ml) and 0.1% of ferric chloride (0.5 ml), and the absorbance was measured spectrophotometrically at 700 nm.

**Chain-breaking activity (CBA)**

The CBA was based on the method of Brand-Williams et al. (1995) ▶ with slight modification. The CBA was expressed by the reaction rate k and calculated by the following equation:

 Abs^-3^- Abs_0_^-3^= -3kt

Where Abs_0_ is initial absorbance, Abs is absorbance at increasing time, (t), and the reaction rate was expressed as k. Antioxidant activity was reported as (-Abs^-3^/min/mg extract).

**Quantity of** **malondialdehyde (MDA)**

Quantification of MDA was conducted according to the combined method of Chawla et al. (1976) ▶. 0.1 ml of sample extract was added to 2 ml of trichloroacetic acid, 2 ml of thiobarbituric acid solution, and 1.9 ml distilled water. This mixture was then placed in a boiling water bath at 100°C for 10 min. After cooling, it was centrifuged at 3000 g for 20 min and absorbance of the supernatant was then measured at 532 nm using UV-Vis spectrophotometer. Quantity of MDA was expressed as µg MDA/ g extract.

**DPPH radical scavenging activity**

The measurement of DPPH radical scavenging activity was carried out according to the method of Barros et al. (2007) ▶. The reduction of DPPH radicals was determined by measuring the absorption at 517 nm. The radical scavenging activity was calculated as a percentage of DPPH discoloration using the following equation:

DPPH radical scavenging % = [(A_0_ – A_1_)/A_0_] × 100

Where A_0_ is the absorbance of the DPPH solution and A_1_ is the absorbance of the sample. 

**Nitric oxide radical inhibition assay (NO°)**

The inhibition of NO° can be estimated by the use of GriessIllosvoy reaction (Garrat, 1964 ▶). In this investigation, GriessIllosvoy reagent was modified using 0.1% of naphthylethylenediaminedihydrochloride instead of 5% 1-napthylamine. The absorbance of solutions was measured at 540 nm against the corresponding blank solutions using the following formula:

Nitric oxide radical scavenging = A_blank_ _ A_sample_ × 100 / A_blank_

**Hydrogen peroxide radical inhibition assay (H**_2_ **O**_2_ **)**

The method described by Ruchet al. (1989) ▶ was used to determine the H_2_O_2 _scavenging ability of extracts. H_2_O_2 _scavenging capacities of the extracts were calculated using the formula:

H_2_O_2 _radical scavenging % = [(A_Blank_ – A_Sample_)/A_Blank_] × 100

**Superoxide radical inhibition assay (O**_2_^-^ **)**

The method described by Jing et al. (1995) ▶ was used to determine O_2_^- ^radical scavenging activity of samples. Briefly, 1 ml of extract was added to 9 ml of 5 mM Tris-HCl buffer (pH 8.2). 40 µl of 4.5 mM pyrogallol was added to the mixture. The mixture was shaken and after 3 min just a drop of ascorbic acid (0.035%) was added to it. The absorbance of the reaction mixture was measured at 420 nm after 5 min (Similar concentration extract was used as the blank to eliminate interference). O_2_^-^radical scavenging activity was expressed by the oxidation degree of a test group in comparison to that of the control. The percentage of scavenging effect was calculated using the following equation:

O_2_^-^ radical scavenging % = [A_0_-(A_1_-A_2_)/A_0_] ×100

Where A_0_ is the absorbance of the Tris-HCl buffer with pyrogallol, A_1_ is the absorbance of the extract addition, and A_2 _is the absorbance of blank extract.

**Statistical analysis**

All experiments were performed in triplicate (n=3) and results were expressed as mean±SEM. Statistical analyses were carried out with (SPSS package version 17.0) using one-way analysis of variance (ANOVA). Significant differences were calculated according to the Tukey’s test. Correlation analysis of the results was performed in SPSS and significant difference was statistically considered at the level of p<0.01.

## Results

**Figure1:**
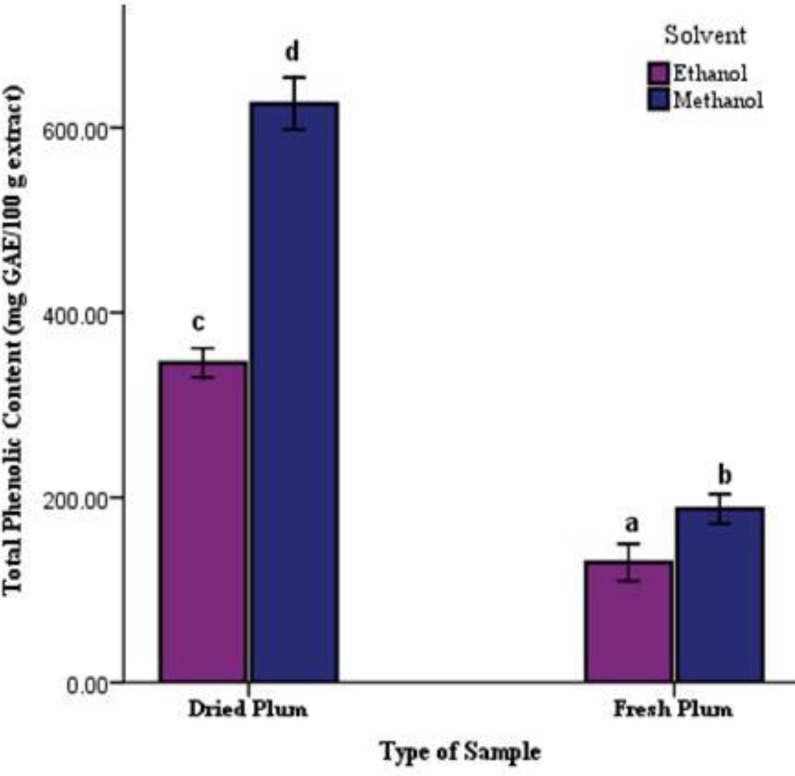
Total Phenolic Content (TPC) of dried and fresh samples of plum in methanolic and ethanolic solvents. Data are means of three replicates with standard errors (Mean±SE, n=3). Columns with the same letters are not significantly different at p<0.01.

The TPC content of samples ranged from 129.93±10.02 to 625.93±14.08 mg GAE/100 g extract. The highest content was recorded in the methanolic extract of dried sample and the lowest was related to the ethanolic extract of fresh sample (Figure 1). 

**Figure2:**
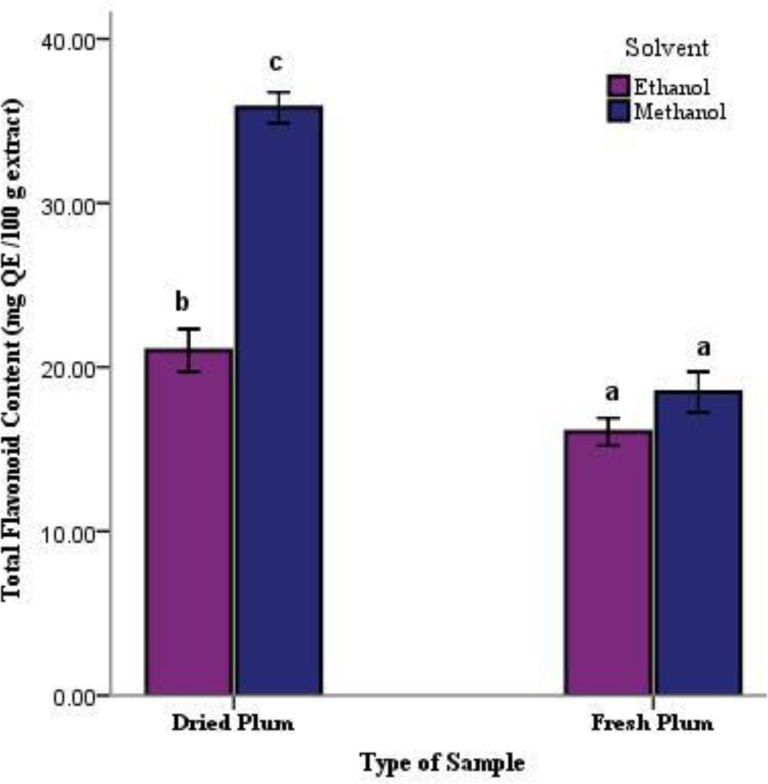
Total Flavonoid Content (TFC) of dried and fresh samples of plum in methanolic and ethanolic solvents. Data are means of three replicates with standard errors (Mean±SE, n=3). Columns with the same letters are not significantly different at p<0.01.

While the TFC content of samples ranged from 16.06±0.041 to 35.81±0.47mg QE/100 g extract, the maximum and minimum values were related to the methanolic extract of dried sample and the methanolic extract of fresh sample, respectively (Figure 2). The TAC ranged from 2.67±0.08 to 16.64±0.58 mg α-tocopherol/ g extract. Most of the TAC was observed in the methanolic extract of dried sample (Table 1).

**Table 1:** Total Antioxidant Capacity (TAC), Reducing Power Assay (RPA), Chain Breaking Activity (CBA), and Quantity of Malondialdehyde (MDA) of dried and fresh samples of plum

**Solvent**	**Type of sample**	**TAC (mg α-tocopherol /gextract)**	**RPA (700 nm)**	**CBA (-Abs-3 /min/mg extract)**	**MDA (µg MDA/g extract)**
**Eethanolic extract**	Dried Plum	11.32 ± 0.44***	1.06 ± 0.02	10.39 ± 2.03	77.70 ± 4.80***
	Fresh Plum	2.67 ± 0.08	0.82 ± 0.01***	1.36 ± 0.06	253.15 ± 16.25***
**Methanolic extract**	Dried Plum	16.64 ± 0.58***	1.08 ± 0.01	33.35 ± 8.32**	24.77 ± 1.02***
	Fresh Plum	3.22 ± 0.16	1.00 ± 00	4.96 ± 0.48	194.25 ± 3.96***

Data are means of three replicates (Mean±SE, n=3). Statistical differences between the data of each extract with those of three other extracts :

**: p<0.01,

***: p<0.001.

The amount of RPA was measured spectrophotometrically at 700 nm. The values obtained were in the range of 0.82±0.01 for ethanolic extract of fresh sample to 1.08±0.01 for methanolic extract of dried sample (Table 1). The results of CBA were ranged from 1.36±0.06 to 33.35±8.32 –Abs^-3^/min/mg extract which the highest rate was also seen in the methanolic extract of dried sample (Table 1). The MDA content was measured using thiobarbituric acid test. The results ranged from 24.77±1.02 to 253.15±16.25 µg MDA/ g extract. In this parameter, the highest value was found in the ethanolic extract of fresh sample and the lowest value was recorded in the methanolic extract of dried sample (Table 1).

DPPH, NO, H_2_O_2_, and O_2_^-^radicals inhibition percentages were measured to assay the antiradical activity of extracts (Table 2).The highest DPPH radical scavenging activity was detected in the methanolic extract of dried sample with 87.94%. The highest NO radical scavenging activity (82.45%) was found in the methanolic extract of dried sample too. Table 2 shows that the methanolic extract of fresh sample had the highest H_2_O_2 _(52.97%) and O_2_^-^(92.00%) radical inhibition percentages. Table 3 shows the results of correlations between all of the parameters. The highest correlation (r^2^=0.977) was found between the TPC and TFC which was showed statistically significant differences (p<0.01).

**Table 2:** 2,2-Diphenyl-1-Picrylhydrazyl (DPPH), Nitric Oxide (NO), Hydrogen Peroxide (H_2_O_2_), and Superoxide (O_2_^-^) radicals scavenging activity of dried and fresh samples of plum

**Solvent**	**Type of sample**	**DPPH (%)**	**NO° (%)**	**H**_2_ **O**_2_ ** (%)**	**O**_2_^- ^ **(%)**
**Ethanolic extract**	Dried Plum	79.78 ± 1.34	76.02 ± 2.15	27.92 ± 1.45***	41.70 ± 1.72***
	Fresh Plum	49.10 ± 1.24***	8.51 ± 1.09**	52.97 ± 1.62	82.95 ± 1.24
**Methanolic extract**					
	Dried Plum	87.94 ± 0.81	82.45 ±1.67	38.97 ± 1.39	62.94 ± 1.72***
	Fresh Plum	62.40 ±1.08***	39.60 ± 1.16**	48.36 ± 0.45	92.00 ± 2.49

Data are means of three replicates with standard errors (Mean±SE, n=3). Statistical differences between the data of each extract with those of three other extracts:

**: p<0.01,

*** : p<0.001.

**Table 3:** Pearson’s correlation coefficients for quantitative determinations in both dried and fresh samples of plum

	TFC	TAC	RPA	CBA	MDA	DPPH	NO	H_2_O_2_	O_2_^-^
TPC	0.977^**^	0.973^**^	0.760^*^^*^	0.909^**^	-0.923^**^	0.922^**^	0.866^**^	-0.547ns	-0.542ns
TFC		0.908**	0.686*	0.917**	-0.835**	0.840**	0.765**	-0.368ns	-0.-361ns
TAC			0.772**	0.861**	-0.957**	0.951**	0.913**	-0.693*	-0.710**
RPA				0.661*	-0.850**	0.925**	0.944**	-0.699*	-0.511ns
CBA					-0.770**	0.786**	0.722**	-0.324ns	-0.340ns
MDA						-0.971**	-0.964**	0.806**	0.739**
DPPH							0.991**	-0.773**	-0.693*
NO°								-0.832**	-0.727**
H_2_O_2_									0.934**

Total Phenolic Content (TPC), Total Flavonoid Content (TFC), Total Antioxidant Capacity (TAC), Reducing Power Assay (RPA), Chain Breaking Activity (CBA), Malondialdehyde (MDA), 2,2-Diphenyl-1-Picrylhydrazyl (DPPH), Nitric Oxide (NO), Hydrogen Peroxide (H_2_O_2_), and Superoxide (O_2_^-^) radicals.A95% confidence interval. ns: no significant,

*: significant at p<0.05,

**: significant at p<0.01.

## Discussion

Plums may be a source of compounds beneficial for human health. As far as antioxidants are concerned, this is associated with a very topical problem of prevention of tumorous conditions (neoplasia) (Chun and Kim, 2004 ▶). The phytochemicals responsible for the antioxidant capacity of fruit are mainly due to phenolic acids and flavonoid compounds (Cao et al., 1997 ▶). Phenolic compounds of prunes consist mainly of chlorogenic acid, neochlorogenic acid, caffeic acid, coumaric acid, rutin (Donovan et al., 1998 ▶), and proanthocyanidin (Kimura et al., 2008 ▶). Total phenolic contents of different plum cultivars have been reported between 282-922 mg/100 g of fruit (Siddiq, 2006 ▶). Of course, the composition of the fruits may have differences due to the growing conditions such as soil, geographical, and environmental conditions during the fruit development, degree of maturity at harvest, and genetic differences (Agata et al., 2009 ▶).

The main phenolic compounds responsible for the pigmentation in plums arecyanidin 3-rutinoside, cyanidin 3-glucoside, and peonidin 3-rutinoside (Kim et al., 2003 ▶). In this study, the TPC of 100 g extract ranged from 129.93 to 625.93 mg GAE and the TFC ranged from 16.06 to 35.81mg QE/100 g extract that in both parameter, dried and fresh samples of methanolic extract and ethanolic extract showed statistically significant differences. (p<0.01, Figures 1 and 2). The results are in agreement with some previous findings stating that deep colored fruits and vegetables are good sources of phenolics including the flavonoids (Cieslik et al., 2006 ▶). Among all of the parameters, the highest correlation (r^2^= 0.977) was found between the TPC and TFC which was significantly different at a level of p<0.01 (Table 3).

At the same time, the TPC and the TAC of plums are relatively high compared with other species of fruit species (Cevallos-Casals et al., 2002 ▶). Moreover, TPC and TAC of prunes were found to be higher than other dry fruits including dates, figs, and raisins (Wu et al., 2004 ▶). Our results showed that the methanolic extract of dried sample and the ethanolic extract of fresh sample were characterized by the highest and lowest total antioxidant capacity, respectively. In this parameter, dried and fresh samples of methanolic extract and ethanolic extract showed statistically significant differences, too (p<0.0001, Table 1). Some authors have reported a direct correlation between TAC and TPC (Ferreira et al., 2007 ▶). In case of European genotype of plums Vasantha Rupasinghe et al. (2006) ▶ found a strong correlation (r^2^ = 0.960) between TAC and TPC. Our results herein were higher than those reported elsewhere (r^2^ = 0.973) (Table 3). The reducing ability of a compound generally depends on the presence of reductants which have exhibited antioxidative potentiality by breaking the free radical chain and donating a hydrogen atom (Dolatkhani and Jamei, 2013 ▶). In RPA assay, ethanolic extract of fresh Plum with those of three other extracts had significant difference at a level of p<0.0001. A strong correlation between the content of TPC and RPA was found in the phenolic extracts of hull and shell of almond (*Rosaceae* family) as reported by Jahanban-Isfahlan et al. (2010 ▶). In our study, the relationship was strong enough (r^2^ = 0.760), too. In CBA assay, a strong correlation coefficient (r^2^ = 0.909) was seen between CBA and TPC of extracts of plum (Table 3).Aboutthis parameter, only the methanolic extract of dried Plum showed significant differences (p<0.01). Thiobarbituric acid test is used to measure the secondary product of oxidation such as aldehyde and ketone (Farag et al., 1989 ▶). The present study showed that in this parameter, the lowest MDA content was detected in the methanolic extract of dried sample that shows this extract has the lowest lipid peroxidation. There was a negative correlation coefficient (r^2^ = -0.923) between MDA content and TPC (Table 3).

DPPH assay is one of the most widely used methods for screening antioxidant activity of plant extracts (Nanjo et al., 1996). DPPH is relatively stable and hence it is a less reactive free radical, so it can be reduced primarily by more reactive reducing components such as phenolic substances (Stratil et al., 2007 ▶). All of the assessed sample extracts revealed a reduction in stability and purple-colored radical DPPH^°^ into the yellow-colored DPPH^°^-H. Moreover, highly significant relationships (p<0.01) were also obtained between DPPH and TPC (r^2^ = 0.922). The obtained results indicated that samples with higher TPC had the strongest free radical scavenging effect. Nitric oxide is the product of nitroprussid reaction with oxygen to form nitrite radicals. Phenolic extracts, as antioxidant compounds, compete with oxygen to combine with nitric oxide and tend to reduce nitrite radical formation significantly, causing transformation of nitric oxide to its reducing products (Marcocci et al., 1994 ▶). The radical scavenging activity of nitric oxide depends upon the extract concentration (Kumaran and Karunakaran, 2007 ▶). In this study, only the fresh samples showed significant differences (p<0.01, Table 2). Moreover, we observed direct and positive correlation (r^2^ = 0.866) between NO and TPC (Table 3). Similar results have also been reported previously on some other *Rosaceae* family plant. Our results support this assumption that dry fruits had higher antioxidant activity than fresh fruits probably due to their low moisture content (Vijaya Kumar Reddy et al., 2010 ▶). Drying process increases the antioxidant activity due to non-enzymatic reaction products, called melanoidins. In prunes, polyphenols contribution to antioxidant activity of prunes is only about 23% of the total antioxidant activity (Madrau et al., 2010 ▶).

H_2_O_2 _is poorly reactive in aqueous solutions at physiological concentrations and is toxic to cells at 10-100 μ levels, and can cross biological membranes rapidly to form cytotoxic hydroxyl radicals (Siriwardhana and Shahidi, 2002 ▶). Reactive free radicals, such as O_2_^- ^and peroxy radical (ROO^.^), are extremely reactive and are known to be a biological product in reducing molecular oxygen (Williams and Jeffrey, 2000 ▶). However, the results about the H_2_O_2 _and O_2_^-^radicals (in comparison with all other parameters) were inversed. Interestingly, the highest scavenging percentage of H_2_O_2 _and O_2_^- ^radicals were obtained in the fresh samples. Two lignin glucosides have been isolated from fresh plum, which have good oxygen radical absorbance activity (Kikuzaki et al., 2004 ▶).

Finally, this research demonstrates that the dried samples of plum contain high levels of TPC, TFC, TAC, RPA, CBA, and MDA content as well as DPPH and NO radicals scavenging activity in comparison with fresh samples. It seems that the dried samples had higher antioxidant activity probably due to their low moisture content and non-enzymatic reaction products. On the other hand, fresh samples of plum are more successful in collecting H_2_O_2 _andO_2_^- ^radicals than dried types. Maybe some of the compounds which have significant role in the inhibition of oxygen radicals may have been destroyed in dried samples. Furthermore, when comparing solvents, it was found that methanol is better for extraction procedure than ethanol. Therefore, these results suggest that plums in both fresh and dried samples can serve as a good source of natural antioxidants and antiradicals. Therefore, it could potentially be considered as a functional food or functional food ingredient. Consequently, simultaneous use of both types of samples can lead to effective antioxidant and antiradical capacity.
